# A case report of a brain herniation secondary to cryptococcal meningitis with elevated intracranial pressure in a patient with Human Immunodeficiency Virus/Acquired immunodeficiency syndrome (HIV/AIDS)

**DOI:** 10.1016/j.idcr.2022.e01554

**Published:** 2022-07-02

**Authors:** Nehemias Guevara, Abdulrasheed Akande, Mailing Flores Chang, Jane Atallah, Carol Epstein

**Affiliations:** a Department of Medicine, Internal Medicine, St. Barnabas Hospital Health System, The Bronx, NY 10457, United States; b Department of Infectious Disease, Internal Medicine, St. Barnabas Hospital Health System, The Bronx, NY 10457, United States

**Keywords:** Meningitis, Cryptococcal meningitis, intracranial pressure, brain herniation, HIV, AIDS

## Abstract

**Background:**

Cryptococcal meningitis is a major opportunistic infection in individuals with HIV. The worldwide annual incidence is estimated to be approximately one million cases per year, with the most significant burden in sub-Saharan Africa. HIV-associated cryptococcal meningitis continues to have a high mortality rate despite widespread availability and use of HAART.

**Case:**

36-year-old male with a past medical history of AIDS and a CD4 count of 35 cells/mm3 presented with altered mental status initially thought to be related to using crystalline methamphetamine as reported by EMS. However, a lumbar puncture performed in the emergency department showed elevated CSF opening pressure of 29 cmH2O and positive CSF and serum cryptococcal antigen. The patient was admitted and commenced treatment according to the current IDSA guideline but continued to have waxing and waning mental status. On the fourth day of admission, he complained of headache, had a witnessed seizure, and was taken emergently for a CT scan of the brain, which was negative for any acute intracranial process, but suffered a cardiac arrest before it could be done. He was intubated and transferred to the intensive care unit. CT brain follow-up showed anoxic encephalopathy, development of marked cerebral edema, and complete effacement of the basilar cisterns, suggestive of downward transtentorial herniation; he continued to deteriorate and expired on the seventh day of admission.

**Objectives:**

1. Describe a case of brain death secondary to increased intracranial pressure due to cryptococcal meningitis in a patient with HIV/AIDS.
2. Explain the mechanisms of elevation in intracranial pressure in patients with cryptococcal meningitis.
3. Discuss the options for managing elevated intracranial pressure in patients with cryptococcal meningitis.
4. Create awareness in the medical community about the importance of prompt and efficient management of increased intracranial pressure in patients with cryptococcal meningitis.

**Conclusion:**

This case highlights the importance of aggressive management of elevated intracranial pressure in cryptococcal meningitis. It reiterates the need for more data regarding the optimal timing and frequency of therapeutic lumbar puncture and the use of temporary lumbar drainage catheters and ventriculostomy to manage this potentially fatal complication.

## Introduction

Cryptococcus is an encapsulated, environmental yeast with two main species responsible for most infections in humans [1] *Cryptococcus neoformans and Cryptococcus gattii* are transmitted via inhalation of the fungus and spread hematogenously with a preference for the central nervous system where they cause cryptococcal meningoencephalitis [1]. *C. gattii* classically causes illness in immunocompetent individuals and was associated with an outbreak of cryptococcosis on Vancouver Island [2] *Cryptococcus neoformans* is a leading cause of infection in immunocompromised hosts. Risk factors include HIV infection, solid organ transplantation using immunosuppressants, sarcoidosis, cirrhosis, and systemic lupus erythematosus [3].

Cryptococcal meningitis due to Cryptococcus neoformans is a major opportunistic infection in individuals with HIV/AIDS, particularly if the CD4 count is less than 100 cells/mm3 [4].

The condition is more prevalent in sub-Saharan Africa but occurs even in developed countries like the United States. The worldwide annual incidence is estimated to be approximately one million cases per year, with the most significant burden in sub-Saharan Africa [1]. Mortality from HIV-associated cryptococcal meningitis remains high despite the widespread availability of HAART [5]. In the hospital, mortality from cryptococcal meningitis is estimated to be between 30% - 50% despite treatment with antifungal agents [6]

A common life-threatening complication of cryptococcal meningitis in HIV patients is raised intracranial pressure (ICP) greater than 25cmH2O. Elevated ICP may be asymptomatic at the time of diagnosis or may be associated with symptoms like headache, vomiting, confusion, loss of vision, hearing impairment, or even death [7] *.* One of the key principles in the treatment of cryptococcal meningitis is the management of elevated ICP, as it has been shown in studies to be an important determinant of treatment outcome [8]. Rolfes et al. reported that therapeutic lumbar punctures were associated with a 69% relative improvement in survival in 248 patients with HIV-associated cryptococcal meningitis in Uganda and South Africa. Several other studies have reported a survival benefit with aggressive management of elevated ICP until pressures have normalized. *(Rolfes* et al.*, 2014a)* Despite these findings and treatment guidelines recommending aggressive ICP management in cryptococcal meningitis, there is no consensus regarding the optimal timing and frequency of therapeutic lumbar puncture [4], [8].

We report the fatal case of a patient with HIV-associated cryptococcal meningitis and raised ICP and examine the role of frequent therapeutic lumbar punctures in the management of elevated ICP which is a cause of significant morbidity and mortality in cryptococcal meningitis.

## Case narrative

36-year-old male with a past medical history of the human immunodeficiency virus (HIV) with acquired immunodeficiency syndrome (AIDS) (poor adherence to antiretroviral therapy (HAART)), major depressive disorder, and unspecified psychotic disorder comes to the emergency department (ED) after being found with altered, signs on arrival to ED were blood pressure of 158/93 mmHg, heart rate (HR): 86 beats/minute, respiratory rate (RR): 18 breaths/minute, O2 saturation 96% on room air, temperature 97.9 F. On physical examination, pupils 3 mm reactive to light bilaterally, no cervical adenopathies, lungs were clear to auscultation, heart sounds were normal, no murmurs, abdomen soft and non-tender, moving all extremities with no focal deficits.

**Image 1:**
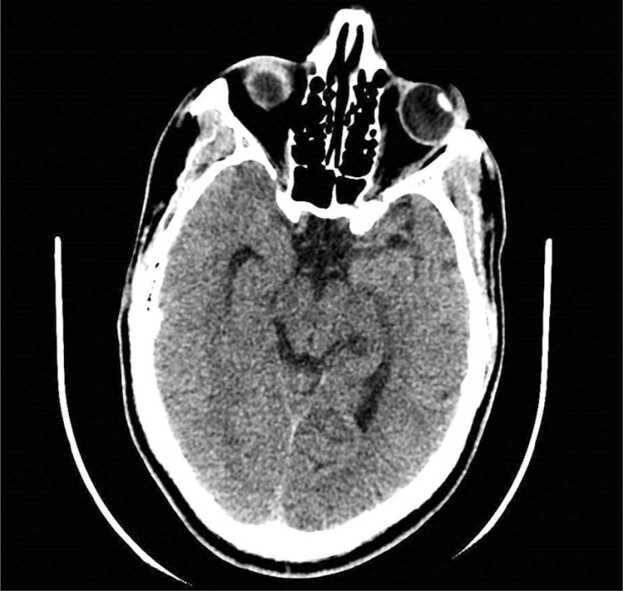
Initial CT brain.

Social history was notable for crystal meth use. Initial blood work was remarkable for leukopenia, mild hyponatremia, and mild elevation of transaminases. (Table 1). CT brain was negative for any acute intracranial abnormality. (Image 1) After 12 h of observation in the ED patient had worsening agitation, groaning, and speaking nonsensical words with a mild grade fever of 100.9 F and sinus tachycardia for which lumbar puncture was done (Table 2) opening pressure was 29 mmHg, serum cryptococcal antigen and RPR positive as well as blood culture positive for cryptococcus neoformans (Table 3). The patient was started on liposomal amphotericin B + flucytosine. Cryptococcal meningitis and cryptococcemia diagnoses were made.

**Table 1:** Laboratory data.

Variable	On admission	Reference range
White cell count	3.8	4.2–9.110*3/uL
Neutrophils	53.6%	34.0–67.9%
Lymphocytes	22.7%	21.8–53.1%
Monocytes	12.0%	5.3–12.2%
Eosinophils	0.0%	0.8–7.0%
Hemoglobin	13.9	13.7–17.5 gm./dL
Hematocrit	42.6	40.1–51.0%
Platelet count	206	150–450 10*3/uL
MCV	88.9	79.0–92.2 fL
MCH	29.0	25.7–32.2 pg
MCHC	32.6	32.3–36.5 gm/dL
Sodium	133	135–145 mEq/L
Potassium	3.8	3.5–5.3 mEq/L
Chloride	98	96–108 mEq/L
Glucose	123	70–99 mg/dL
Calcium	9.4	9.2–11.0 mg/dL
Creatinine	1.0	0.6–1.2 mg/dL
ALT	37	4–36 IU/L
AST	46	8–33 IU/L
Bilirubin Total	0.7	0.1–1.2 mg/dL
TSH	0.95	0.34–5.60 u[IU]/mL
Magnesium	1.9	1.3–2.1 mEq/L

**Table 2:** Cerebrospinal fluid analysis.

Variable	Result	Reference range
Color	Pink	Colorless
Appearance	Clear	Clear
Xantocromía	Negative	Negative
WBC	10	0–5 / mm3
RBC	8165	0–0 / mm3
Neutrophil	3	0–6%
Lymphocyte	84	40–80%
Monocyte	7	15–45%
Macrophage	6	%
Other:		
Cryptococcus	Present	
Glucose	41	40–70 mg/dL
Protein	78	15–45 mg/dL
Cryptococcal antigen	Positive – 1:1280	
Culture CSF	Cryptococcus neoformans	
Acid fast bacilli culture	No growth	
Fungal culture	No fungus isolated	
India Ink preparation	Positive for cryptococcus neoformans	
VDRL	Non reactive

**Table 3:** Other laboratory results.

Variable	Result	Reference range
Serum cryptococcal antigen	Positive – 1:640	
RPR	Reactive – 1:32	
LDH	937	100–190 IU/L
Procalcitonin	0.07	0.00–0.08 ng/mL
Absolute CD4 helper count	35	359–1519 /uL
Blood culture	Yeast, Cryptococcus neoformans	
Urine toxicology:		
Barbiturates	Negative	
Benzodiazepines	Negative	
Cocaine	Negative	
Opiates	Negative	
THC	Negative	

During hospitalization, patient developed encephalopathy leading to seizures and bradycardia delaying therapeutic lumbar punction, ultimately causing cardiac arrest twice with achievement of return of spontaneous circulation within 4 min and 2 min, respectively.

Further neurological evaluation was remarkable for pupils 7 mm bilaterally, not reactive to light, corneal reflex absent bilaterally, gag reflex absent, no movement of extremities to deep painful stimulation, flaccid tone, DTRs absent, plantars mute.

**Image 2:**
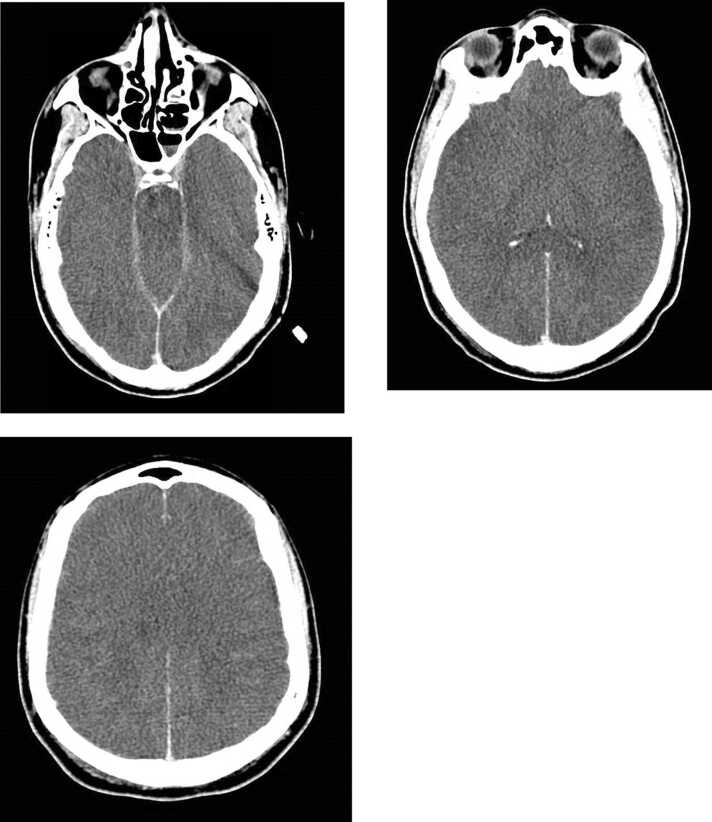
Follow up CT Brain.

CT brain was repeated, which showed diffuse cerebral edema with loss of the cervical pattern, suggestive of anoxic encephalopathy and development of marked cerebral edema, with complete effacement of the basilar cisterns, suggestive of downward transtentorial herniation. (Image 2) Brain death determination test was by clinical examination and apnea testing, which confirmed death by neurological criteria.

## Discussion

Cryptococcus neoforman is a ubiquitous encapsulated yeast-like fungus. Risk factors for developing symptomatic cryptococcosis include HIV infection with low CD4+ lymphocyte count (< 50 cells/mm3), malignancy (chronic leukemia, lymphoma), steroids treatment, organ transplantation, and sarcoidosis [9].

Cryptococcal meningitis is one of the most prevalent opportunistic infections in patients with HIV/AIDS, aiming for approximately 220,000 cases of cryptococcal meningitis worldwide per year, resulting in nearly 181,000 deaths, even though most of these cases are reported in sub-Saharan Africa, have been demonstrated a prevalence of cryptococcal antigenemia to be 2.9% in the USA [10], [11]

The mechanism for which C. neoformans increase the intracranial pressure is still poorly understood, however, it has been shown that it is caused due to the significant burden of yeast and polysaccharides that plugs the arachnoid villi [12], [13], [14]. Therefore, there exists a positive correlation between the number of organisms and the size of the polysaccharide capsule with the increase in the ICP [14], [15]. Factors predictive of high mortality during antimycotic therapy include 1) cerebrospinal fluid (CSF) cryptococcal antigen titer greater than 1:1024; 2) abnormal mental status, 3) and a CSF leukocyte count <20 cells/µL with high ICP [16], [17] Our patient presented with all of these characteristics, arriving with altered mental status, high intracranial pressure (290), antigen titer 1:1280 with low WBC (10) in the CSF.

Furthermore, an increase in intracranial pressure in patients with cryptococcal meningitis is still one of the most deadly complications, and even though antifungal treatment has improved, intracranial pressure treatment is still a gray area.

Current treatment for cryptococcal meningitis is divided into 2 phases, first is induction with amphotericin B (0.7–1 mg/kg/d) plus flucytosine (100 mg/kg/d for two weeks) and then followed by fluconazole (400 mg/d) for a minimum of 10 weeks, after ten weeks with fluconazole this can be reduced 200 mg/d, depend on patient clinical status, alternative regimens are available as well, and in a patient with renal impairment lipid formulations of Amphotericin B are available and preferred [18], [19].

Our patient was started on Amphotericin B liposomal (due to AKI at admission) and flucytosine.

Use of steroids, acetazolamide, and mannitol in the setting of increased ICP due to cryptococcal meningitis is not recommended [20], [21], [22], [23], [24].

Multiple clinical trials are ongoing regarding the use of steroids in cryptococcal meningitis, with promising results, but at the moment are still not recommended as an adjunct therapy [25], [26], [27].

Management of ICP in patients with cryptococcal meningitis is still a challenge; with high mortality, and morbidities. It’s associated with multiple complications that vary in severity from severe to mild and can be presented at the beginning of the disease or well as later.

Complications such as strokes [28], blindness which are caused either by the direct spread and secondary inflammation to the optic nerves (optic neuritis), or compressive optic neuropathy secondary to raised ICP [29].

Brain herniation, is the most deadly complication of cryptococcal meningitis, even after the resolution of the disease, some studies have shown the patients that who presented cryptococcal meningitis with an elevation of ICP increase the overall risk of dying one year after the first presentation, and become an independent factor of poor prognosis [30], [31], [32].

Studies have shown the mortality of CM in patients with HIV/AIDS can be 50% in the first two weeks after the diagnosis [33], this could be associated with the increase in the burden of the cryptococcus after the initiation of the treatment and subsequent increase in ICP. Therefore, close monitoring of any signs of clinical deterioration, or change in mental status is recommended, especially if the open pressure of the ICP was increased since the beginning of the CM [33], [34].

Current treatments for high ICP include serial LP [35], [36], and shutting. Lumbar puncture as part of treatment is based on clinical manifestations and has shown survival improvement in different studies [34], [37], [38]. The benefits of aggressive LP as a treatment overweight the risk of brain herniation, which has been described as a possibility in LP in the setting of high ICP in patients with CM [38], [39]. Lumbar puncture is recommended when the open pressure is more than 250, and the goal should be to keep open pressure <200 this has been described to decrease mortality and improve outcomes. The frequency is unclear and should be based on the clinical assessment [34], [33], [31], [40], [12].

Alternative therapeutics approaches such as CSF shunting through a lumbar drain or ventriculostomy are available and should be considered for patients when LP is not well tolerated, or symptoms worsen even with repetitive LP, sudden decline in mental status, or evidence of hydrocephalus [41], [42], [43], [44].

Unfortunately, our patient died, and the second LP never was possible to be done. Brain death was determined by clinical examination and apnea testing.

The initiation of the HAART therapy is recommended to be started four to six weeks after antifungal agents are initiated, and at that time CSF should be clear [45], [46].

This has been proved to prevent immune reconstitution inflammatory syndrome (IRIS) and decrease mortality six months after initiating HAART [40], [47], [48]

## Conclusion

This case highlights the importance of aggressive management of elevated intracranial pressure in cryptococcal meningitis. It reiterates the need for more data regarding the optimal timing and frequency of therapeutic lumbar puncture and the use of temporary lumbar drainage catheters and ventriculostomy to manage this potentially fatal complication.

## Funding

This case report has not been financial supported by any entity.

## Ethics approval

The study was approved by the ethical committee of St. Barnabas Hospital, City University of New York, in New York, US.

## Consent

Informed written consent was obtained from the patient for the publication of this case report and the accompanying images.

## Author statement

All the authors have worked equally in this case report.

## Competing interests

None of the authors has a financial and non-financial competing interest.
